# Physical Activity and Sports Participation among Adolescents: Associations with Sports-Related Knowledge and Attitudes

**DOI:** 10.3390/ijerph19106235

**Published:** 2022-05-20

**Authors:** Leila Oja, Jaanika Piksööt

**Affiliations:** National Institute for Health Development, 11619 Tallinn, Estonia; jaanika.piksoot@tai.ee

**Keywords:** sports training, sports-related knowledge, physical activity, personal attitudes, adolescents

## Abstract

The impact of physical activity and sport on the development of adolescents’ knowledge and attitudes has not been studied sufficiently. We assume that young people with more knowledge of sports will continue to be physically active on their own. The aim of this study is to identify the differences in the sports and physical activity-related knowledge and attitudes of adolescents who engage in organised sports training and those who do not. A total of 1033 6th-grade students from (aged 12.7 ± 0.4 years) 52 schools in Estonia were involved in the study. Logistic regression analysis was used to examine the associations between sports-related knowledge and attitudes towards students participating in organised sports training. The main reasons which hindered adolescents from being active were a lack of suitable equipment, being weaker than others, and laziness. Knowledge of physical activity, higher perceived benefits, and lower barriers to physical activity were the predictors contributing to adolescents’ participation in sports training. The results demonstrated that physically active students have better knowledge of sports and physical activity. Students participating in organised sports training have strong positive benefits from being regularly physically active and feel that sports training maintains their health.

## 1. Introduction

Physical activity leads to numerous health and psychological benefits. Besides improved physical and mental health and better well-being, it also supports age development and relationships with peers and parents. The extent to which physical activity and participation in sports training contributes to the development of a student’s knowledge and attitudes about sports has not been extensively studied.

Studies show that regular physical activity promotes growth and development and has multiple benefits for physical, mental, and psychosocial health that undoubtedly contribute to learning [1]. It is also known that physical activity affects academic achievement and cognitive performance [2]. Most scientific articles confirm the ongoing decline in physical activity, but it is not yet clear what are the factors related to this change [3,4]. The literature on physical activity shows that this change has multidimensional characteristics: psychological, social, cultural, environmental, and behavioural [5,6].

Knowledge about a behaviour plays a significant role in encouraging people to change their health behaviours and knowledge of health-related physical activity is linked with increased physical activity among children, adolescents, and youth [7,8,9]. With increasing age, contribution to active play decreases and formal sport increases [10].

WHO guidelines (2021) recommend that children and adolescents should do an average of at least 60 min per day of moderate to vigorous-intensity, mostly aerobic, physical activity, throughout the week [11]. Vigorous-intensity aerobic activities, as well as muscle and bone-strengthening activities, should be performed at least 3 days a week [12]. Recent global data reveal no overall improvement in global levels of physical activity participation over the last two decades—27% of adults and 81% of adolescents do not meet the recommendations for aerobic exercise [13,14].

In order to improve children’s physical activity, two approaches are mainly used: physical education lessons or school-based interventions [2,15,16,17,18,19] and activities outside of school [20,21]. It has been shown that school-based interventions can have a positive effect on knowledge of nutrition and physical activity among elementary school children [22,23]. Also, the physical activity-related knowledge provided by peers can have positive effects by increasing knowledge and promoting physical activity among adolescent girls [24].

It is stated that to increase physical activity levels, it is important to determine adolescents’ perceived benefits, barriers, and cues to engaging in physical activity [25]. It has also been found that in order to adopt a physically active lifestyle, it is critical to understand the principles and concepts of health-related fitness [26]. An important factor which helps to improve students’ physical activity is students’ knowledge of sports, physical activity, and a healthy lifestyle. There is reason to believe that the physical skills and experience that have been acquired with the help of this knowledge are stable and will help students to make better use of these skills in adulthood. Research has found that the most common reason for adolescents’ physical activity is enjoyment, whereas the most common barrier is wanting to do other things with one’s time. “Having a friend to exercise with” was the most helpful cue to becoming involved in physical activity for both males and females [25]. It is known from previous research that young people who experience more barriers have fewer opportunities to become active [27], and the perceived barriers are the main inverse predictor of physical activity [3]. Identification of the perceived barriers to physical activity is important in order to improve our understanding of physical activity behaviour among adolescents and to develop effective physical activity interventions for youth.

So far, it is not entirely clear whether participation in organised sports training is associated with students’ sports knowledge and attitudes towards being physically active such as the barriers and benefits. The aim of this study is to find out how much physical activity and sports, among other benefits including physical, mental, or social, relate to young people’s knowledge of physical activity and sports, and how accurate this knowledge is. We also explain young people’s attitudes towards being physically active.

## 2. Methods

### 2.1. Sample

The research was conducted among 1033 students aged 11–13 (mean age 12.7 ± 0.4 years) in the 2012/2013 school year. The sixth-grade schoolchildren from four of Estonia’s largest counties—in the northern, southern, eastern, and western parts of the country—took part. As a result, 52 (of 78 selected schools) schools agreed to participate in the study. During the data collection phase, the non-response was monitored by the lack of balance index. This index helps to balance the set of respondents with the set of auxiliary variables that were specified by strata definition and the belonging of the school to the health-promoting movement. So, the final response rate was 66.7% in each group defined by the auxiliary variables. In this paper, the data of 917 respondents (453 boys and 464 girls) were used, as they had completed the physical activity questionnaire. The students were divided into two groups according to their participation in organised sports training. The Ethics Committee of the Faculty of Medicine, University of Tartu, approved this study.

### 2.2. Measures

*Physical activity*. The students’ physical (in)activity was assessed by three questions including daily moderate physical activity, participation in regular organised sports training, and screen-time duration (watching TV, using the internet, playing inactive computer games). The wording of the questions is shown in Table 1.

*Knowledge.* The respondents’ knowledge of physical activity and sports was assessed by four questions (Table 1). For the physical activity question, it was possible to obtain a total of 3 points (3 correct answers). For the sports questions, respectively, 2 points per each correct answer (endurance, strength, skilfulness) were obtained. Open-ended answers to the sports questions were coded as follows: 0 = wrong answer; 1 = one correct answer; 2 = two or more correct answers. The total knowledge score of 9 points was calculated as follows: physical activity 3 points, endurance 2 points, strength 2 points, and skilfulness 2 points.

*Attitudes.* From the respondents’ personal attitudes, the perceived barriers and benefits of being regularly physically active were assessed. Ten statements were presented about both the benefits and barriers that might affect the respondents’ regular physical activity of at least two times a week (Table 2). Participants indicated how much they agreed with each of these statements on a scale from (1) *Do not agree* … to (5) *Totally agree*.

### 2.3. Statistical Methods

Descriptive statistics included calculating mean and standard deviations (m ± SD), proportions, and variable distributions for the entire sample and gender-stratified. To determine statistical differences between groups, ANOVA and Chi-square statistics were used.

Logistic regression analysis with activity level as a binary dependent variable (participates in sports training vs. does not participate in sports training) was assayed to verify associated knowledge, barriers, and benefits to physical activity. For the regression analysis, total scores were calculated for variables used in the model. At first, simple logistic regression analysis was used for assessing how each individual factor is associated with participation in sports training. Next, multiple regression analysis was used for clarifying adjusted associations of all factors, including socio-demographic variables—sex (boys vs girls), school’s instructional language (Estonian vs non-Estonian), assessment of family’s economic situation (on a scale of 1 = very bad … 5 = very good). As a result, adjusted and unadjusted odds ratios (OR) and 95% confidence intervals (CI) were presented.

The statistical analysis was performed by using IBM SPSS software (versions 14 and 22 for Windows; Chicago, IL, USA); *p* values < 0.05 were denoted as statistically significant.

## 3. Results

The study revealed that 63% of children took part in organised sport training (Table 3). A total of 12.5% of school children were physically active for at least 60 min a day seven days a week and 33% on five or more days a week. Most of the children, 87%, watch TV, play inactive computer games, or use the internet for up to 2 h per day. All analysed variables did not differ statistically between genders.

Table 4 shows the mean total knowledge scores according to the physical activity classification categories for boys, girls, and the total. According to the results, the children that participated in organized sports training achieved significantly higher scores in sports-related knowledge. The knowledge score was also higher for respondents that were physically active for at least 60 min on five or more days a week when compared to less-active children. On the other hand, there were no significant associations between the screen-time duration and the sports-related knowledge of the respondents.

According to the adjusted regression analysis, perceived benefits of physical activity (AOR = 1.64, CI = 1.32–2.03) was the strongest predictor for participation in organised sports training (Table 5). Other personal factors such as knowledge of physical activity (AOR = 1.15, CI = 1.05–1.27) and perceived barriers to physical activity (AOR = 0.46, CI = 0.38–0.56) were also statistically significantly associated with sports participation. Also, the results confirmed the already well-known outcome that a family’s economic situation is significantly associated with children’s participation in organised sports training (AOR = 1.28, CI = 1.01–1.63).

The results of the perceived benefits and barriers comparing students participating in organised sports training and students who do not participate in organised sports training are shown in Table 6. Students who participate in sports training are more likely to feel that being physically active is fun (m = 4.6; SD = 0.7) when compared to students who do not participate in sports training (m = 4.0; SD = 1.1; F = 76.3; *p* = 0.00). Also, students who participate in sports training are proud of it (F = 69.4; *p* = 0.00) and they find that sports training helps to keep them healthy (F = 51.0; *p* = 0.00).

The largest differences in the barriers to being regularly physically active between the student groups were the lack of suitable equipment (F = 106.6; *p* = 0.00) and rating themselves weaker than the others (F = 92.4; *p* = 0.00).

## 4. Discussion

This analysis confirmed the understanding that physical activity has a positive effect on increasing students’ knowledge in the field of sports and movement. The results of this study indicate that participating in sports training has a positive effect on knowledge of physical activity and sports. Recent studies have shown that this is not always valid and there could be differences in knowledge and perception. The importance of knowledge in sports and fitness has also been stressed in the context of the school system. The results of Williams and co-authors [28] underscore that more efforts are needed to teach adolescents health-related fitness concepts and potentially mandate a concept-based physical education course in schools. With increasing age, knowledge related to physical activity and healthy behaviour concepts improves, but previous studies have yet to show a high enough level of knowledge in children necessary for enhancing physical activity experience and skills [19,29]. Brusseau and co-authors [29] indicated in a study that assessed children aged 5–13 years, that the students had minimal success and demonstrated little understanding of physical activity and health behaviour concepts and also expressed some misconceptions. This study showed that, in general, the level of knowledge related to sports and physical activity was average. Most of the children (73%) cannot identify sports events which develop physical skilfulness; there was, however, a better understanding of strength and endurance.

About 20% of the respondents in our study were able to give two or more correct answers to the knowledge items, and more than half of them participated in sports training. The total average knowledge score was high in children who were physically active at least 60 min per day on five or more days per week. Roth and Stamatakis [30], using the 2007 Health Survey from England, examined whether knowledge of the guidelines is linked to physical activity levels for children aged 11–15. For girls, meeting the guidelines was associated with a knowledge of them, but it was weak among boys, for whom meeting the guidelines was associated only with having a white ethnicity.

A small increase in physical activity or the implementation of physical activity interventions, both in school and outside of school, provides evidence of an increase in knowledge or understanding [15,17,18]. Consequently, it is expedient to continue to focus on guiding students towards sports.

Inadequate daily physical activity in children continues to decline, despite the WHO’s recommendation to be physically active at a moderate to vigorous level for at least 60 min daily [11]. In our study, 12.5% of students fulfilled these recommendations. These results are similar to the results of the HBSC study report during the same period, 14% [31]. The current analysis showed that approximately 63% of respondents participated in sports training; however, the five days or more of physical activity (60 min daily) was met only by 33% of young people. Despite efforts during recent decades to promote physical activity among adolescents, 25% of students still do not know the actual recommended amount of physical activity per day.

The results showed that students’ participation in sports is significantly associated with the perceived benefits and barriers to physical activity as well as their knowledge of sports. A significant barrier is the inability of young people to exert themselves because among the main barriers to being physically active are “laziness” and “it is physically very hard”. According to the results, it could be recommended to offer suitable training according to the capabilities of adolescents. However, it is significant that low physical activity is also caused by a lack of financial resources and time or friends to exercise with.

## 5. Conclusions

In conclusion, it should be noted that knowledge of physical activity and sports is better among students who participate in organised sports training and/or who are physically active for at least 60 min per day five days a week. Students who participate in organised sports training perceive fewer barriers and recognise the additional benefits of being physically active, including being proud of it and maintaining their health. Therefore, it can be suggested that, in addition to practical skills, it is important to pay more attention to increasing students’ sports-related knowledge through physical education programs in schools.

## Figures and Tables

**Table 1:** Student-level items used to measure physical activity and knowledge.

Topic	Description of Questions
Physical (in)activity	How often are you physically active at least 60 min per day, so that you are sweating or panting? Response categories: (1) *Rarely or never* … (5) *Every day.* Do you participate regularly in sports training (sports or dance classes, or some other active hobbies and activities) at least 2 times a week? Response categories: *Yes/No*. How many hours in the school day do you usually watch television, play computer games, or are on the internet? Response categories: (1) *Not at all* … (5) *More than 4 h*.
Knowledge about physical activity	How often each week do you think is beneficial to your health to participate in sports or active movement (according to general recommendations)? Select all correct answers. Response categories: At least 60 min of moderate physical activity in a day At least 30 min of high-intensity physical activity at least three times per week At least 10,000 steps a day Two hours of high-intensity physical activity each day 20 min of moderate physical activity two times a week
Knowledge about sports	What do you think are the sports events that develop endurance? Give examples. What do you think are the sports events that develop strength? Give examples. What do you think are the sports events that develop skilfulness? Give examples.

**Table 2:** Student-level statements used to measure barriers and benefits of being regularly physically active.

Benefits of Regular Physical Activity	Barriers to Regular Physical Activity
It is fun	Do not have time
I am proud of it	Other duties
Helps to find new friends	Laziness
I can spend more time with my friends	Living far from sports facilities
Helps me stand out	It is expensive
Keeps me in good health	Do not have suitable equipment
Improves my sporting achievements	Weaker than others
Keeps me healthy	It is physically very hard
Helps me avoid getting fat	Get tired easily
Gives me energy	Health problems

**Table 3:** Descriptive statistics of the respondents’ physical activity (PA). Statistical significance was calculated by using Chi-square analysis.

PA Categories	Boys n = 453		Girls n = 464		Total n = 917		χ^2^	Sig.
	n	%	n	%	n	%		
Participating in organised sports training	279	61.6	296	63.8	575	62.7	0.48	0.49
PA at least 60 min on 5 or more days a week	157	35.2	146	31.7	303	33.4	1.22	0.27
Screen time up to 2 h a day	388	88.0	389	85.7	777	86.8	1.03	0.31

**Table 4:** Mean knowledge score (±SD) according to physical activity (PA) classification categories for boys and girls. Statistical significance was calculated by using analysis of variance.

PA Categories	Boys		Girls		Total			
	n	m ± SD	n	m ± SD)	n (%)	m ± SD	F	Sig.
Participating in organised sports training								
Yes	279	4.2 ± 1.7	296	4.5 ± 1.7	575 (63)	4.4 ± 1.7	37.9	0.00
No	174	3.5 ± 1.9	168	3.8 ± 1.8	342 (37)	3.6 ± 1.9		
PA at least 60 min a day								
Up to 4 days a week	289	3.8 ± 1.8	314	4.2 ± 1.7	603 (67)	4.0 ± 1.8	4.9	0.03
5 or more days a week	157	4.2 ± 1.7	145	4.4 ± 1.8	302 (33)	4.3 ± 1.8		
Screen time								
Up to 2 h a day	388	4.0 ± 1.8	389	4.3 ± 1.7	777 (87)	4.2 ± 1.8	2.2	0.14
More than 2 h a day	53	3.9 ± 1.8	65	3.9 ± 1.8	118 (13)	3.9 ± 1.8		

**Table 5:** Logistic regression analysis of the socio-demographic and personal factors associated with participation in organised sports training.

Factors	Univariate Model				Multivariate Model			
	OR	95% CI		Sig.	Adjusted OR	95% CI		Sig.
		Lower	Upper			Lower	Upper	
Socio-demographic factors								
Gender (Boys vs girls)	0.91	0.70	1.19	0.49	1.14	0.83	1.57	0.42
School language (Estonian vs non-Estonian)	1.68	1.26	2.24	0.00	1.30	0.92	1.83	0.13
Family’s economic situation ^1^	1.38	1.12	1.70	0.00	1.28	1.01	1.63	0.05
Personal factors								
Knowledge of PA and sports ^1^	1.27	1.17	1.38	0.00	1.15	1.05	1.27	0.00
Perceived barriers to PA ^1^	0.39	0.32	0.47	0.00	0.46	0.38	0.56	0.00
Perceived benefits of PA	2.14	1.77	2.60	0.00	1.64	1.32	2.03	0.00

^1^ Inserted into regression models as a covariate.

**Table 6:** Adolescents’ perceived benefits and barriers to physical activity (m ± SD) according to their participation in organised sports training. Statistical significance was calculated by using analysis of variance.

Answer Choices (1—*Do not agree* ... 5—*Totally agree*)	Does Not Participate in Sports Training				Participates in Sports Training				F	Sig.
	n	%	m	SD	n	%	m	SD		
**Benefits of being regularly physically active**										
It is fun	226	70	4.0	1.1	487	90	4.6	0.7	76.3	0.00
I am proud of it	207	65	3.8	1.1	447	83	4.4	0.9	69.4	0.00
Helps to find new friends	172	54	3.7	1.2	407	75	4.2	1.0	43.3	0.00
I can spend more time with my friends	197	61	3.8	1.1	421	78	4.2	1.0	35.7	0.00
Helps me stand out	144	45	3.4	1.2	309	57	3.8	1.1	19.6	0.00
Keeps me in good shape	236	74	4.1	1.0	456	85	4.5	0.8	35.9	0.00
Improves my sports achievements	221	69	4.0	1.1	470	87	4.5	0.8	47.7	0.00
Keeps me healthy	237	73	4.1	1.0	468	87	4.5	0.8	51.0	0.00
Helps me avoid getting fat	221	69	4.0	1.1	427	79	4.3	1.0	22.1	0.00
Gives me energy	207	64	3.8	1.2	409	77	4.2	1.0	31.3	0.00
**Barriers to being regularly physically active**										
Do not have time	176	54	3.4	1.4	197	35	2.7	1.5	50.6	0.00
Other duties	176	54	3.4	1.4	224	40	2.9	1.4	29.9	0.00
Laziness	131	40	3.0	1.4	107	19	2.2	1.3	76.5	0.00
Living far from sports facilities	113	34	2.8	1.4	102	18	2.1	1.3	58.0	0.00
Do not have suitable equipment	88	27	2.6	1.4	42	8	1.7	1.0	106.6	0.00
Weaker than others	70	21	2.4	1.3	40	7	1.7	1.0	92.4	0.00
It is physically very hard	61	19	2.3	1.3	36	6	1.6	1.0	70.8	0.00
Get tired easily	83	25	2.5	1.4	71	13	1.9	1.1	61.5	0.00
Health problems	46	14	2.1	1.3	58	10	1.7	1.1	17.9	0.00
It is expensive	72	22	2.4	1.4	48	9	1.7	1.1	69.1	0.00

## Data Availability

The data presented in this study are available on request from the corresponding author.
